# Retrospective Cohort Study on Potential Risk Factors for Repeated Need of Dental Rehabilitation under General Anesthesia in a Private Pediatric Dental Practice

**DOI:** 10.3390/children9060855

**Published:** 2022-06-08

**Authors:** Viktoria Karl, Konstantin Johannes Scholz, Karl-Anton Hiller, Isabelle Tabenski, Frederike Schenke, Wolfgang Buchalla, Christian Kirschneck, Katrin Bekes, Fabian Cieplik

**Affiliations:** 1 Department of Conservative Dentistry and Periodontology, University Hospital Regensburg, 93053 Regensburg, Germany; viktoria-karl@web.de (V.K.); konstantin.scholz@ukr.de (K.J.S.); karl-anton.hiller@ukr.de (K.-A.H.); wolfgang.buchalla@ukr.de (W.B.); 2 Private Pediatric Dental Practice “Die Kinderzahnärzte Dr. Schenke & Kolleginnen”, 93051 Regensburg, Germany; tabenski@diekinderzahnaerzte-west.de (I.T.); schenke@diekinderzahnaerzte-west.de (F.S.); 3 Department of Orthodontics, University Hospital Regensburg, 93053 Regensburg, Germany; christian.kirschneck@ukr.de; 4 Department of Pediatric Dentistry, University Clinic of Dentistry, Medical University of Vienna, 1090 Vienna, Austria; katrin.bekes@meduniwien.ac.at

**Keywords:** general anesthesia, pediatric dentistry, children, risk factors

## Abstract

The need for dental rehabilitations under general anesthesia (DRGAs) is continuously increasing, particularly for dental treatment of children. The present retrospective cohort study aimed to investigate potential risk factors for repeated need of DRGA in a cohort of patients from a private pediatric dental practice. Demographic and anamnestic data, dental status, and treatments performed during DRGA were retrospectively analyzed from the electronic dental charts of 1155 children that received at least one DRGA between October 2016 and December 2021. The median age of all children was 5 years at time of their first DRGA. The rate of repeated DRGAs was 9%. Patients with repeated need of DRGA were significantly younger at time of their first DRGA and revealed significantly more often a history of preterm birth and current use of a baby bottle as compared to patients with only one DRGA. There were significantly fewer treatments (regardless of type) in the second DRGA than at the first. Within the limitations of this study, young age at first DRGA, a history of preterm birth, and current use of a baby bottle may be risk factors for repeated need of DRGA. The search for effective strategies to minimize the repeated need for DRGA in children remains critical.

## 1. Introduction

Untreated caries of deciduous teeth is among the most prevalent non-communicable diseases and affects 532 million children worldwide [1]. According to data from the German Oral Health Study series [2,3,4], the caries experience has been declining continuously over the last two decades from an average of 1.7 decayed, missing, or filled (DMF) teeth of twelve-year old children in 1997 [2] to 0.7 DMF teeth in 2005 [3] and 0.5 DMF teeth in 2014 [4]. However, some children are still severely suffering from caries because caries prevalence is not distributed equally among children and strongly depends on the respective socio-economic background [4]. Accordingly, the mean dmft of 3-year-old children was 0.48 in 2016, while those 3-year-olds with caries experience had a mean dmft of 3.6 [5]. In addition, about three-quarters of the decayed primary teeth in the 3-year-olds were not restored [5].

While most children can be successfully treated with the aid of local anesthesia, general anesthesia (GA) can be indicated for performing dental treatments in children with insufficient compliance, especially in children of young age, in children suffering from general diseases or disabilities, or in children with extensive treatment need, e.g., due to severe early childhood caries (ECC) [6,7,8,9,10,11]. In Germany, alternatives to GA are not very widespread for patients with extensive treatment needs in pediatric dental practice. For example, Splieth et al. reported in 2020 that 71% of general dental practitioners referred children to specialized pediatric dentists for treatment under GA, whereas 91% did not refer children for treatment under nitrous oxide sedation [12], although the latter is better accepted by parents [13]. On the other hand, oral sedation by means of midazolam is mostly limited to short treatments, e.g., rehabilitation of single quadrants, and poses the risk of decreasing cooperativeness on multiple sessions [14]. Furthermore, midazolam is not universally offered by pediatric dentists, most likely due to safety issues [15]. Therefore, the number of dental rehabilitations under general anesthesia (DRGA) in children is constantly increasing [6,8,16,17,18,19], and many authors recorded repeated need of DRGAs [19,20,21,22,23,24,25,26,27]. Accordingly, previous studies have reported rates of 9% for receiving two or more DRGAs in the US [19], 10.8% in Canada [23], and 11% in Germany [26].

In this light, it must be kept in mind that severe complications can occur from GA in children [28]. For instance, Campbell et al. reported an overall complication rate of 1.1% when examining 351 DRGA cases, with occurrence of two cases of postextubation croup, and one case each of mild intraoperative bronchospasm and intraoperative bradycardia [29]. Therefore, it is important to investigate potential risk factors for repeated DRGAs in order to potentially limit the numbers of GAs due to dental treatment need in children. Previous studies have identified insufficient adherence to preventive recall appointments and oral hygiene recommendations (e.g., fluoride use) following the first DRGA as potential risk factors for repeated DRGAs [19,20]. Furthermore, poor treatment planning and a too conservative treatment strategy at the first DRGA have also been discussed to contribute to repeated need of DRGAs [24,30,31,32]. However, most of these studies have been conducted in academic hospital settings, which may differ from private practices in some points, such as a substantially higher proportion of children with comorbidities (ASA scores II and III) [16,19,33]. On the other hand, there is much less data from private pediatric dental practice settings [11].

Therefore, the aim of this retrospective cohort study was to investigate a patient cohort that received DRGAs in a private pediatric dental practice in Germany with regard to potential risk factors for repeated need of DRGAs. The null-hypothesis tested was that there were no differences between the demographic and anamnestic data, dental status, and performed treatments during DRGA between the children who received DRGA once and those who received at least two DRGAs.

## 2. Materials and Methods

### 2.1. Study Design

The present study was designed as a retrospective cohort study. The objective was to investigate potential risk factors for repeated need of DRGAs in a cohort of patients from a private pediatric dental practice.

The study design was approved by the institutional review board of the University of Regensburg, Germany (reference: 21-2726-104) in accordance with the 1964 Helsinki Declaration and its later amendments and comparable ethical standards. The study was registered at the German Clinical Trials Register (DRKS00028511).

### 2.2. Patient Cohort

All patients from the patient pool of the private pediatric dental practice “Die Kinderzahnärzte Dr. Schenke & Kolleginnen” (Regensburg, Germany), who had received DRGAs from the establishment of the practice in October 2016 until end of December 2021, were screened for inclusion in this study. The inclusion criteria applied were that patients had to be less than 18 years old and had received at least one DRGA. There were no exclusion criteria. All data were collected from the electronic dental charts by one single examiner (V.K.).

### 2.3. Demographic and Anamnestic Data

The following socio-economic data were collected: Age, gender, insurance status (statutory health insurance, private health insurance, social welfare), DRGAs of siblings, and the socio-economic status of each parent (seeking work, unskilled worker, job that requires training, academics), whereby the higher socio-economic status of both parents was considered. Data on medical history considering known general diseases or conditions, American Society of Anesthesiologists physical status classification system (ASA), medications, allergies, previous treatment under GA (and corresponding general medical or dental indication), and preterm birth anamnesis were collected from the dental charts, as well as data on fluoridation measures before DRGA (tablets, salt, toothpaste, gel, no fluoridation measures). Furthermore, indication for DRGA (lack of compliance, age in relation to clinical treatment need, known general diseases or conditions), and information about current use of a baby bottle and current breastfeeding habits were gathered.

### 2.4. Dental Status and Performed Treatments during DRGA and Post-Operative Recall

The numbers of decayed (d; D), missing (m; M), and filled (f; F) teeth were recorded at each DRGA and the resulting dmft (primary dentition) and DMFT (mixed or permanent dentitions) scores were calculated. ECC was classified according to the nomenclature proposed by Wyne [34].

Furthermore, numbers of direct (composite or compomer restorations) or indirect (stainless steel crowns or all-ceramic crowns) restorations, endodontic treatments (pulpotomies, root canal treatments), extractions, and fissure-sealings were recorded. Attendance of post-operative follow-up within four weeks after DRGA and number of preventive recalls within the first post-operative year (≤2; >2) were assessed.

### 2.5. Data Analysis

Frequency tables were generated and medians with 1st and 3rd quartiles were calculated. Statistical evaluation was performed using non-parametric statistical procedures (Mann–Whitney *U* or χ^2^ tests) on a significance level α = 0.05. All statistical analyses were performed using SPSS version 26 (SPSS Inc., Chicago, IL, USA).

## 3. Results

### 3.1. Patient Cohort

A total of 1155 patients who received DRGA in the private pediatric dental practice from October 2016 to the end of December 2021 could be included into this study. From these 1155 patients, 1051 (91%) received one DRGA and 104 (9%) received at least two DRGAs (94 patients receiving two, 9 receiving three, and 1 receiving four DRGAs). The median (1st; 3rd quartile) time intervals were 17.8 (11.8; 25.0) months between first and second DRGA and 11.0 (9.4; 19.4) months between second and third DRGA.

### 3.2. Demographic and Anamnestic Data at Time of First DRGA

All demographic and anamnestic data for the included 1155 patients at time of their first DRGA is shown in Table 1.

The median age of all patients was 5 years. 161 patients (13.9%) were younger than 3 years, 596 (51.6%) between 3 and 6 years, 380 (32.9%) between 6 and 12 years, and 18 (1.6%) at least 12 years old. There was a slight predominance of male patients (53.3%). Most of the patients were classified as ASA I (86.6%), while 11.3% were ASA II and 2.1% ASA III. 13.5% of the patients had known general diseases or conditions, whereby the most common were neurodermatitis (4%), asthma (1.7%), and autism (1%). For 3.8% of the patients, regular intake of medications was recorded, most frequently cetirizine (0.4%), L-thyroxine, and salbutamol (both 0.3%). 7.9% of the patients reported allergies, mostly pollen (3%) and food allergies (2.7%), but 1.3% also reported allergies toward clinically relevant drugs such as penicillin (1%) or ibuprofen (0.3%).

Indication for DRGA was mostly due to insufficient compliance for regular dental treatment (52.2%) or low age in relation to clinical treatment need (47.2%). Most of the patients had public health insurance (97.6%). The socio-economic status of the parents was distributed between academics (8.2%), jobs that require training (47.6%), and unskilled workers or people seeking work (23.9%), while in 20.3% the socio-economic status was not classifiable from the dental charts. 5.4% had a history of preterm birth, 87.7% had a previous visit at a dentist, 22.7% a previous GA, mostly due to nasal polypectomy (3.7%) or due to previous DRGA in another practice (4.8%). 14.5% of the patients had siblings that also had received DRGA. Before the DRGA, 32.4% used no fluoridation measures at all, while fluoridated toothpaste was used by 46.4%, fluoridated gel by 3.8%, fluoridated salt by 12.3%, and fluoride tablets by 3.3%. Current breastfeeding was reported in 2% of the patients and current use of a baby bottle was reported in 16.1%.

When comparing the 1051 patients who received one DRGA to those 104 who received at least two DRGAs (Table 1), the latter group was significantly younger at their first DRGA (*p* < 0.001), comprised significantly more patients with history of preterm birth (*p* = 0.013) and reported significantly more often the current use of a baby bottle (*p* < 0.001).

### 3.3. Dental Status and Treatments during DRGA and Post-Operative Follow-Up

Table 2 shows the dental status of all patients at their DRGAs. At their first DRGA, the 1155 patients had median dmft of 8 for primary dentitions (mainly based on a median of 8 decayed primary teeth), a median DMFT of 9 for mixed dentitions (mainly based on a median of 7 decayed primary teeth), and a median DMFT of 4.5 for permanent dentitions (mainly based on a median of 4.5 decayed permanent teeth). Regarding ECC, 22.8% of the patients were classified as ECC type I, 29.6% as ECC type II, and 7.3% as ECC type III.

Table 3 shows the performed treatments during DRGA as well as the attendance to post-operative follow-up and preventive recall appointments. With regard to treatments during the first DRGA, direct restorations with resin-based composites or compomers were performed in 90.1% of the patients (median number of 7 per DRGA), while stainless steel or all-ceramic crowns were performed in 62.3% of the patients (1 in median per DRGA) and fissure sealings in 42.5% of the patients. 56.3% of the patients received pulpotomy treatment (1 in median per DRGA), 9% received root canal treatment, and 62.9% received extractions (1 in median per DRGA). 82.2% of the patients attended the post-operative follow-up within four weeks after DRGA, but 42.7% did not attend any of the following preventive recall appointments during the first post-operative year.

When comparing the dental status and performed treatments between the 1051 patients that received one DRGA and the 104 receiving at least two DRGAs, the latter received significantly more root canal treatments (*p* < 0.001) and there also was a tendency for fewer extractions (*p* = 0.051). Furthermore, there was a significant difference regarding attendance of preventive recall appointments (*p* = 0.026).

When comparing the dental status and performed treatments in the first and the second DRGA of the 104 patients, significantly fewer fissure sealings (*p* = 0.007), direct restorations (*p* < 0.001), crowns (*p* = 0.003), pulpotomies (*p* < 0.001), and root canal treatments (*p* < 0.001) were performed in the second DRGA. Furthermore, there was a significant difference regarding attendance of preventive recall appointments (*p* = 0.001).

## 4. Discussion

This study aimed to investigate potential risk factors for repeated DRGAs in a large patient cohort of 1155 children and adolescents that received at least one DRGA in a private pediatric dental practice setting. For this purpose, demographic and anamnestic data, dental status, and performed treatments during DRGA as well as attendance of post-operative follow-ups and preventive recall appointments were investigated.

The median age at the first DRGA (5 years) and the slight predominance of male patients (53.3%) are in line with earlier studies [7,11,19,20,25,26]. Likewise, the rate of 9% for repeated DRGAs is in a similar range as reported previously [19,20,26,35]. The median time interval between first and second DRGA was slightly shorter in the present cohort (median of 17.8 months) as compared to other studies, which reported mean intervals of 22 [20], 26.1 [36], or 34.4 months [25], which may be due to the different settings (university hospital vs. private practice).

Pediatric dentistry departments of university hospitals often receive referrals of patients with severe underlying diseases or conditions that contradict DRGAs at private practice settings [7,19,33]. For instance, in the study by Delfiner et al., about half of the patients receiving DRGA in a university setting exhibited severe comorbidities and corresponding ASA scores II, III, and IV [7]. On the other hand, two German studies showed similar proportions of medically compromised children as in the present study, irrespective of whether they were treated at private practice or university [11,20]. As also described by Takriti et al. [11], there were only very few children reporting regular intake of medications, and allergies were also just recorded in a small proportion of the patients. The indication of DRGA was mainly based on lack of compliance or low age in relation to clinical treatment need. Likewise, Savanheimo and Vehkalahti reported insufficient compliance, dental fear, and excessive need for treatment as most common reasons for DRGA in healthy children [35]. Notably, about 15% of the patients of the present study had siblings that also had received DRGA, which is in line with König et al. (25% siblings with history of DRGA) [20], and consistent with the polarization of caries experience reported in the 5th German Oral Health study [4]. When estimating socio-economic status of the parents based on their current jobs, only slightly more than half of the families were from an academic background or had jobs that require training. This is also reflected in the low rate of patients with private health insurance of less than 2%, in contrast to epidemiologic data showing that in the Eastern Bavarian region (where the practice is located) about 20% of the patients have private health insurance [37]. Accordingly, Ramdaw et al. showed that low parental socio-economic status was associated with increased need for DRGA [10]. These aspects clearly show that the cohort of children included in the present study is a particularly vulnerable group with an increased caries risk at a young age and consequently an early need for dental treatment and particularly for DRGA.

About one third of the patients’ parents reported that no fluoridation measures were applied, but it should be considered that parent reporting regarding fluoridation frequently may not be reliable. For instance, Martins et al., recently reported significant differences between parent-reported (39%) and actual use (71.4%) of fluoride toothpaste [38]. So, fluoride use may be underestimated, and the parents may have used fluoride toothpaste for their children unwittingly, but the frequency of tooth brushing and fluoride application was not recorded.

The dental status at first DRGA in terms of median dmft or DMFT scores mirrors previous studies in Germany [11,20], while the prevalence of ECC was recorded at 59.7% here, which is quite in the middle between the 14.1% or 20.1% described by Takriti et al. for an academic setting or private practices, respectively, and the 95.7% described by König et al. for an academic setting [20]. This difference may be mainly attributed to different age distributions among these studies with some patients being simply too old (i.e., >71 months of age) to be classified as ECC [39]. As reported in previous studies [11,20,26,36], the treatments performed during the first DRGA mainly comprised direct restorations, stainless steel or all-ceramic crowns, pulpotomies, and extractions, while root canal treatments were performed in less than 10% of the DRGAs. For direct restorations, resin-based composites or compomers were employed, which previously showed no significant differences in survival rates for restoration of class II cavities in primary molars, while there was a tendency for higher survival when the restorations were placed in GA as compared to local anesthesia [40].

When comparing patients who received one DRGA with those who received at least two DRGAs, three aspects were particularly striking. First, patients who received repeated DRGAs were significantly younger at the time of their first DRGA, which seems to be a logical consequence because the younger the age at DRGA, the higher the likelihood of needing another DRGA during the period of age when compliance may still be limited, particularly in case of insufficient adherence to preventive recall appointments. Furthermore, if DRGA is performed before full eruption of all primary teeth, hypomineralized second primary molars (HSPM) indicative for subsequent molar-incisor hypomineralization (MIH) cannot be diagnosed [41,42], potentially making a consecutive DRGA more likely. The number of treatments (regardless of type) was significantly lower in the second DRGA than in the first, which is consistent with the findings of König et al. [20]. Consequently, a less conservative treatment approach favoring extractions of “questionable” teeth may decrease the risk for repeated DRGAs, particularly in young children [24]. Second, the proportion of patients with a history of preterm birth was significantly higher among those with repeated DRGAs. Preterm birth has previously been associated with ECC [43] and with dental caries in preschool children [44], but there are also contradictory results [45]. Primary teeth of preterm-born children may exhibit signs of disturbed mineralization, such as more porosities and a lower Ca/C ratio indicating a lower degree of mineralization, making them more prone for demineralization [46]. Furthermore, behavioral management problems have also been reported to be more common in preterm born children during their pre-school period [47], which may explain the higher need for repeated DRGAs. Third, patients with repeated need for DRGAs reported significantly more often to currently use a baby bottle. ECC has previously been even termed “baby bottle caries” [34] and is strongly associated with use of baby bottles, particularly containing sugary drinks [48].

Attendance at preventive recall appointments may help prevent the repeated need for DRGA [11,20]. In the present cohort, the vast majority of patients attended post-operative follow-up within four weeks of their first DRGA, whereas the rate of attendance to further preventive recall appointments within the first post-operative year was considerably lower. This can be explained, at least in part, by the large catchment area of the private pediatric dental practice in Regensburg in the geographic context of the Eastern Bavarian region (Niederbayern and Oberpfalz). This does not necessarily mean that no further recall appointments took place, but rather that the patients’ parents may have simply preferred to visit their local general dentist rather than incur long distances and additional travel costs to Regensburg, as discussed previously in another context [49]. The significantly lower attendance rate of patients with repeated DRGAs may be explained by the shorter follow-up time in these patients, reflected in the high proportion of patients for whom the first post-operative year had not yet elapsed.

Despite the potential limitations of a retrospective study design, data collection based on electronic dental charts can generally be considered reliable as they are the basis for treatment charges in statutory or private health insurances in Germany. The rather short follow-up time for the later DRGAs (especially those from 2020 or 2021) may lead to an underestimation of the repeated need for a DRGA [19], particularly since some of the children may have changed to another pediatric or general dental practice following their first DRGA. Future (prospective) studies should also include more anamnestic data, especially on oral hygiene behavior, which could be an important risk factor for the repeated need for DRGA.

## 5. Conclusions

The null-hypothesis could be partially rejected, since the present study identified young age at time of first DRGA, history of preterm birth, and current use of a baby bottle as risk factors for repeated need of DRGA in a patient-cohort from a private pediatric dental practice. For preventing repeated DRGAs, it might be worthwhile to consider a less conservative treatment approach in favor of extractions, particularly if children receive their first DRGA at a rather young age. Furthermore, adherence to post-operative recall appointments seems crucial to avoid repeated need of DRGAs. More (prospective) studies are needed to identify further risk factors for repeated DRGAs for finding effective strategies to avoid repeated DRGA in children.

## Figures and Tables

**Table 1:** Demographic and anamnestic data of all included patients at the time of their first DRGA.

	Time of 1st DRGA			Significant Differences *
	**All Patients**	**Patients with 1 DRGA**	**Patients with ≥2 DRGAs**	1 DRGA vs. ≥2 DRGAs
	1155 (100%)	1051 (91%)	104 (9%)	
**Age** [years]	5 (3.7; 6.7)	5.1 (3.8; 6.8)	4.2 (3.1; 5.7)	<0.001 ^a^
**Gender** [%]				
female	46.7	46.8	45.2	– ^b^
male	53.3	53.2	54.8	
**ASA classification** [%]				
ASA I	86.6	86.7	85.6	– ^b^
ASA II	11.3	11.2	12.5	
ASA III	2.1	2.1	1.9	
**Known general diseases or conditions** [%] ^#^				
yes	13.5	13.4	14.4	– ^b^
no	85.0	85.2	83.7	
**Regular intake of medications** [%] ^#^				
yes	3.8	3.6	4.9	– ^b^
no	94.5	94.7	93.3	
**Allergies** [%] ^#^				
yes	7.9	7.7	9.6	– ^b^
no	90.5	90.7	88.5	
**Indication for DRGA** [%]				
lack of compliance	52.2	52.2	52.1	– ^b^
age in relation to clinical treatment need	47.2	47.2	46.9	
known general diseases or conditions	0.6	0.6	1	
**Insurance status** [%]				
statutory health insurance	97.6	97.6	97.1	– ^b^
private health insurance	1.6	1.6	1.9	
social welfare	0.8	0.8	1.0	
**Socio-economic status of the parents** [%]				
academic	8.2	8.2	8.7	– ^b^
job that requires training	47.6	47.7	47.1	
seeking work, unskilled workers	23.9	23.8	25	
not classifiable	20.3	20.4	19.2	
**Preterm birth** [%]	5.4	4.9	10.6	0.013 ^b^
**Previous visit at dentist** [%]	87.7	88	84.6	– ^b^
**Previous GA** [%]	22.7	22.5	25	– ^b^
**Previous DRGA** [%]	5.7	5.6	7.7	– ^b^
**Siblings receiving DRGA** [%]				
yes	14.5	14.2	18.3	– ^b^
no	85.5	85.8	81.7	
**Fluoridation measures before DRGA** [%] ^§^				
toothpaste	46.4	46	52	– ^b^
gel	3.8	3.6	4.8	
salt	12.3	12.5	10.6	
tablet	3.3	3.2	3.9	
no fluoridation measures	32.4	32.5	30.8	
unknown	15.1	15.4	11.5	
**Current breastfeeding** [%]	2	2.2	1	– ^b^
**Current use of baby bottle** [%]	16.1	14.7	30.8	<0.001 ^b^

Depiction of medians (1st quartile; 3rd quartile) or relative proportions. * Statistically significant differences were calculated from pairwise comparisons between patients that received 1 DRGA or ≥2 DRGAs (^a^ Mann–Whitney *U* or ^b^ χ^2^-tests, respectively; α = 0.05). *p*-value, significant (*p* ≤ 0.05); –, not significant (*p* > 0.05). ^#^ Percentages do not add up to 100% due to missing data in <2% of the patients. ^§^ Multiple answers per patient were possible.

**Table 2:** Dental status of all included patients at their respective DRGAs.

		1st DRGA		2nd DRGA	Significant Differences *	
	**All Patients**	**Patients with 1 DRGA**	**Patients with ≥2 DRGAs**	**All Patients**	1 DRGA vs. ≥2 DRGAs	1st vs. 2nd DRGA
	1155 (100%)	1051 (91%)	104 (9%)	104 (9%)		
**Age** [years]	5 (3.7; 6.7)	5.1 (3.8; 6.8)	4.2 (3.1; 5.7)	5.8 (4.5; 7.3)	<0.001 ^a^	n.d.
**dmft primary dentition**	8 (5; 10)	8 (5; 10)	8 (5; 10)	11 (7.5; 14)	– ^a^	<0.001 ^a^
d	8 (5; 10)	8 (5; 10)	7 (5; 10)	4 (1.5; 5.5)		
m	0 (0; 0)	0 (0; 0)	0 (0; 0)	0 (0; 0.5)		
f	0 (0; 0)	0 (0; 0)	0 (0; 0)	6 (3; 9)		
**DMFT mixed dentition**	9 (6; 11)	9 (6; 11)	9 (6; 11)	11 (8; 14)	– ^a^	<0.001 ^a^
d	7 (4; 9)	7 (3.3; 9)	8 (6; 9.5)	3 (2; 5)		
m	0 (0; 0)	0 (0; 0)	0 (0; 0)	0 (0; 0)		
f	0 (0; 2)	0 (0; 2)	0 (0; 1)	7 (3; 8)		
D	0 (0; 2)	0 (0; 2)	0 (0; 0.5)	0 (0; 0)		
M	0 (0; 0)	0 (0; 0)	0 (0; 0)	0 (0; 0)		
F	0 (0; 0)	0 (0; 0)	0 (0; 0)	0 (0; 0)		
**DMFT permanent dentition**	4.5 (2; 12.8)	4.5 (2; 12.8)			n.d.	n.d.
D	4.5 (2; 10)	4.5 (2; 10)				
M	0 (0; 0)	0 (0; 0)				
F	0 (0; 2.5)	0 (0; 2.5)				
**ECC classification** [%]						
no ECC	40.3	41.2	31.7	^§^	– ^b^	n.d.
type I	22.8	22.9	21.2			
type II	29.6	28.6	39.4			
type III	7.3	7.2	7.7			

Depiction of medians (1st quartile; 3rd quartile) or relative proportions. * Statistically significant differences were calculated from pairwise comparisons between patients that received 1 DRGA or ≥2 DRGAs or between data for the 1st and 2nd DRGA for the patients who received ≥2 DRGAs (^a^ Mann–Whitney *U* or ^b^ χ^2^-tests, respectively). *p*-value, significant (*p* ≤ 0.05); –, not significant (*p* > 0.05); n.d., not determined. ^§^ ECC classification was only recorded at the first DRGA.

**Table 3:** Performed treatments in all patients during their DRGAs as well as attendance of post-operative recall.

		1st DRGA		2nd DRGA	Significant Differences *	
	**All Patients**	**Patients with 1 DRGA**	**Patients with ≥2 DRGAs**	**All Patients**	1 DRGA vs. ≥2 DRGAs	1st vs. 2nd DRGA
	1155 (100%)	1051 (91%)	104 (9%)	104 (9%)		
**Fissure sealings**	0 (0; 3)	0 (0; 4)	0 (0; 2)	0 (0; 0)	– ^a^	0.007 ^a^
no	57.5	57.4	58.7	76		
1–2	15.8	14.7	26	16.3		
3–4	19.1	20	10.6	7.7		
≥5	7.6	7.9	4.8	-		
**Direct composite or compomer restorations**	7 (4; 10)	7 (5; 10)	7 (4; 10)	3 (0; 6)	– ^a^	<0.001 ^a^
no	9.9	9.6	12.5	28.8		
1–6	39	39.9	30.8	51		
7–12	39.3	39.2	40.4	19.2		
13–18	10.1	9.9	12.5	1		
≥19	1.6	1.4	3.8	-		
**Stainless steel or all-ceramic crowns**	1 (0; 3)	1 (0; 3)	2 (0; 3)	0 (0; 2)	– ^a^	0.003 ^a^
no	37.7	38.1	33.7	56.7		
1–2	32.6	33	28.8	26		
3–4	19	18.1	27.9	9.6		
5–6	7.3	7.6	3.8	4.8		
≥7	3.5	3.2	5.8	2.9		
**Pulpotomies**	1 (0; 2)	1 (0; 2)	1 (0; 2)	0 (0; 1)	– ^a^	<0.001 ^a^
no	43.7	44.1	40.4	71.2		
1–2	37.1	36.9	39.4	21.2		
3–4	14.8	14.7	16.3	6.7		
≥5	4.3	4.4	3.8	1		
**Root canal treatments**	0 (0; 0)	0 (0; 0)	0 (0; 0)	0 (0; 0)	<0.001 ^a^	<0.001 ^a^
no	91	92.1	79.8	100		
1	4.9	4.7	7.7	-		
2	2.3	1.8	7.7	-		
≥3	1.7	1.4	4.8	-		
**Extractions**	1 (0; 3)	1 (0; 3)	1 (0; 2)	1 (0; 3)	(0.051) ^a^	– ^a^
no	37.1	36.3	45.2	30.8		
1–3	42.6	43	38.5	57.7		
4–6	14.5	14.6	13.5	10.6		
≥7	5.9	6.2	2.9	1		
**Post-operative follow-up** [%]						
attendance within four weeks after DRGA	82.2	82.5	78.8	73.1	– ^b^	– ^b^
**Preventive recall appointments** [%]						
no preventive recall appointment	42.7	43.3	36.5	26.9	0.026 ^b^	0.001 ^b^
≤2 within first post-operative year	27.5	26.3	40.4	36.5		
>2 within first post-operative year	12.5	12	17.3	7.7		
first post-operative year not expired	17.3	18.5	5.8	25.0		

Depiction of medians (1st quartile; 3rd quartile) or relative proportions. * Statistically significant differences were calculated from pairwise comparisons between patients that received 1 DRGA or ≥2 DRGAs or between data for the 1st and 2nd DRGA for the patients who received ≥2 DRGAs (^a^ Mann–Whitney *U* or ^b^ χ^2^-tests, respectively). *p*-value, significant (*p* ≤ 0.05); –, not significant (*p* > 0.05).

## Data Availability

All data supporting the reported results are available upon request from the corresponding author.
